# Balancing biomass reaction stoichiometry and measured fluxes in flux balance analysis

**DOI:** 10.1093/bioinformatics/btad600

**Published:** 2023-09-27

**Authors:** Axel von Kamp, Steffen Klamt

**Affiliations:** Analysis and Redesign of Biological Networks, Max Planck Institute for Dynamics of Complex Technical Systems, Magdeburg 39106, Germany; Analysis and Redesign of Biological Networks, Max Planck Institute for Dynamics of Complex Technical Systems, Magdeburg 39106, Germany

## Abstract

**Motivation:**

Flux balance analysis (FBA) is widely recognized as an important method for studying metabolic networks. When incorporating flux measurements of certain reactions into an FBA problem, it is possible that the underlying linear program may become infeasible, e.g. due to measurement or modeling inaccuracies. Furthermore, while the biomass reaction is of central importance in FBA models, its stoichiometry is often a rough estimate and a source of high uncertainty.

**Results:**

In this work, we present a method that allows modifications to the biomass reaction stoichiometry as a means to (i) render the FBA problem feasible and (ii) improve the accuracy of the model by corrections in the biomass composition. Optionally, the adjustment of the biomass composition can be used in conjunction with a previously introduced approach for balancing inconsistent fluxes to obtain a feasible FBA system. We demonstrate the value of our approach by analyzing realistic flux measurements of *E.coli*. In particular, we find that the growth-associated maintenance (GAM) demand of ATP, which is typically integrated with the biomass reaction, is likely overestimated in recent genome-scale models, at least for certain growth conditions. In light of these findings, we discuss issues related to the determination and inclusion of GAM values in constraint-based models. Overall, our method can uncover potential errors and suggest adjustments in the assumed biomass composition in FBA models based on inconsistencies between the model and measured fluxes.

**Availability and implementation:**

The developed method has been implemented in our software tool CNApy available from https://github.com/cnapy-org/CNApy.

## 1 Introduction

Several mathematical modeling techniques have been developed to analyze and explore metabolic networks. Constraint-based modeling is one such technique, which, in its basic form, is founded on the network structure (stoichiometry) and linear equality and inequality constraints (Maarleveld *et al.* 2013, Simeonidis and Price 2015). The fundamental assumption of the modeling framework is that the metabolite concentrations are in a steady state. The most commonly used constraint-based method is flux balance analysis (FBA), which searches for metabolic flux distributions that optimize a given linear objective function, such as growth rate maximization (Raman and Chandra 2009, Orth *et al.* 2010). Normally, FBA problems have a solution but may become infeasible when containing contradictory constraints, e.g. due to inconsistent flux values obtained from measurements.

In a previous paper (Klamt and von Kamp 2022), methods to resolve infeasibilities that primarily stem from the integration of inconsistent flux measurements were developed and their practical application demonstrated. The main idea is to allow corrections of the measurements and then to minimize the (weighted) sum of these corrections. Depending on the minimization scheme (linear or quadratic) and weights of the corrections (absolute or relative to the flux values or dependent on measurement accuracy), the solutions have different properties and the applicability of the different schemes/weights to concrete situations was discussed. Furthermore, relationships of the new methods to classic balancing techniques from metabolic flux analysis were clarified.

In the present work, we focus on the additional possibility to adjust the stoichiometry of the biomass (pseudo) reaction, also known as biomass objective function (Feist and Palsson 2010), to resolve infeasible FBA scenarios. As before, we assume that the metabolic model is correct and do not aim to resolve possibly existing modeling errors (e.g. unbalanced reaction equations) with our method. Allowing adjustments of the biomass reaction is relevant for several reasons. First of all, models usually contain one biomass reaction, but in reality, the biomass composition (and thus the stoichiometry of the biomass reaction) can vary with the growth condition (Shahab *et al.* 1996, Pramanik and Keasling 1997, Zhang *et al.* 2016). Also, determination of the biomass composition is, despite support via computational pipelines (Lachance *et al.* 2019), still challenging and can produce different results (Simensen *et al.* 2022). Having a fixed biomass reaction that represents the average composition of the organism is a useful first approximation and appropriate in many situations, but can be overly stringent in certain cases, as we will show in this article. This particularly relates to the growth-associated maintenance (GAM) demand of ATP which is typically integrated in the biomass reaction as an amount of ATP hydrolyzed to ADP providing the energy necessary for reproduction processes such as polymerization of macromolecules (proteins, RNA, DNA) or DNA proofreading. The total amount of ATP (and precursors) needed for growth is then obtained by multiplying the biomass reaction stoichiometry [describing the amounts (usually in mmol) of compounds needed for building 1 g of biomass] with the growth rate. Most FBA models contain additionally a separate ATP hydrolysis reaction (ATP + H_2_O → ADP + Pi + H+) representing the nongrowth-associated ATP maintenance (NGAM) demand, which, as the name indicates, is independent of the growth rate and includes processes such as repair mechanisms or maintaining a constant pH.

GAM can be estimated in two ways: The first method uses known energy requirements for growth processes, e.g. approximately 2 ATP and 2 GTP are required for the polymerization of one amino acid into a protein molecule. For DNA, RNA, and protein synthesis, the GAM for 1 g *E.coli* biomass has been estimated to be 21.970, 0.256, and 0.137 mmol/gDW, respectively (Neidhardt *et al.* 1990). The sum of these values (22.363 mmol/gDW) can be seen as a lower bound for the GAM demand of *E.coli*.

The second method for GAM estimation uses experimental data together with a metabolic model. Basically, the model is set up without any maintenance requirements and is then used to calculate the maximum amount of surplus ATP it can generate under constraints from experimental data (typically comprising substrate uptake, fermentation product excretion, and growth rate). The relation between surplus ATP and the growth rate is then used to estimate GAM. An illustration of this method can be found in Supplementary Figure 7 of Monk *et al.* (2017). There, the calculated maximal ATP production is plotted against the measured growth rate for several sets of measurements. A regression line for this plot is calculated and its slope gives the GAM value estimate. In this particular setting, the NGAM value is also derived from the plot as the *y*-intercept of the regression line. Table 1 shows a selection of GAM estimates for *E.coli* calculated from experimental data using a metabolic model.

**Table 1.** GAM estimates for *E.coli* calculated from experimental data in conjunction with a metabolic model.

Reference	Varma *et al.* (1993)	Feist *et al.* (2007)	Orth *et al.* (2011)	Monk *et al.* (2017)
GAM (mmol/gDW)	23	59.81	53.95	75.38

As can be seen from Table 1, GAM estimates vary significantly, which means that there is a high uncertainty as to what the real value is, which translates to a high uncertainty of predicted metabolic fluxes in FBA studies (Dinh *et al.* 2022). Estimating GAM in this manner is considered to be a good practice (Thiele and Palsson 2010) but can in certain situations lead to problems as will be shown next.

Another reason for allowing adjustments to the biomass reaction derives from the circumstance that models from automatic reconstruction pipelines (Seaver *et al.* 2021) usually contain a rather generic biomass composition (e.g. “gram negative bacterium”). Since it is often not easy to obtain accurate measurements of an organism’s true composition, it can be desirable to have a method available for an automatic adjustment of the composition based on measured metabolic fluxes.

## 2 Materials and methods

### 2.1 Definitions

The nomenclature used herein follows the one of our previous paper (Klamt and von Kamp 2022). We consider a metabolic network with stoichiometric matrix $N\in R^{m\times n}$ consisting of m metabolites and n reactions. We assume that the network is in steady state so that the vector of reaction rates, $r\in R^{n}$, satisfies


(1) Nr=0.

The admissible range of a reaction rate can be constrained with


(2) lbi≤ri≤ubi

to make the reaction irreversible or to integrate a more specific requirement like a substrate uptake limit or some minimum flux, e.g. for the NGAM reaction. Since fixed reaction rates are treated separately (see below), we demand that ${lb}_{i}\neq {ub}_{i}$. General inequality constraints of the form


(3) Ar≤b 

may also be integrated in an FBA problem, e.g. to embed enzyme allocation constraints (Sánchez *et al.* 2017, Bekiaris and Klamt 2020) FBA problems have a linear objective function, such as maximization of the growth rate, which is then optimized subject to constraints (1)–(3):


(4) max cTrs.t. 1–(3) 

Equations (1)–(4) constitute a linear program (LP) which can be solved by various solvers and we assume that it has a feasible solution. This is usually the case for metabolic models without fixed (known) rates.

With F⊂{1…n}, we denote the set of indices of all reactions that have a fixed (known or measured) flux and their corresponding reaction rates are fixed via equality constraints:


(5) ri=fi, ∀ i∊F.

Adding equality constraints (5) to the base system (1)–(3) may now render the FBA problem infeasible and we briefly recapitulate how this situation can be resolved with an LP or a quadratic program (QP). For resolving infeasibility, a correction term (or slack variable) $\delta_{i}∊R$ is added to each of the fixed reaction rates:


(6) ri=fi-δi, ∀ i∊F.

The weighted sum of the squared corrections can then be minimized as a QP


(7) $$\begin{array}{c} min\sum_{i∊F}w_{i}\delta_{i}^{2}.\\ s.t.\;\left(1\right)–\left(3\right)\;\mathrm{and}\;\left(6\right),w_{i}\geq 0 \end{array}$$

where $w_{i}$ is the weight for the corresponding correction$\;\delta_{i}$. Alternatively, one may use separate variables for positive ($\delta_{i}^{+}$) and negative ($\delta_{i}^{-}$) corrections


(8) ri=fi-δi++δi-, ∀ i∊F, δi+≥0, δi-≥0,

and then minimize the weighted sum of these corrections resulting in the LP:


(9) min∑i∊Fwi(δi++δi-).s.t. 1–3, 8, wi≥0

For further details, including the choice of appropriates weights and advantages and disadvantages of the LP and QP variants, we refer to Klamt and von Kamp (2022).

### 2.2 The biomass reaction

The basis of the biomass reaction stoichiometry is the quantities of biomass components needed to build 1 g dry weight (gDW) of cell biomass. Hence, the sum of the component coefficients (mmol/gDW) multiplied by their respective molecular weights (g/mmol) must yield 1 g (Thiele and Palsson 2010). There are two major variants of biomass reactions, depending on their components: often, the components are metabolites [precursors (e.g. pyruvate or acetyl-CoA) or/and monomers such as amino acids or nucleotides as well as cofactors such as ATP and NADPH] that are produced by the elementary reactions of the network. In this case, the biomass reaction may contain several dozen components. In the other case, the components in the biomass reaction represent macromolecules (e.g. protein, DNA, RNA) or complex metabolites (e.g. lipids or vitamins) that are in turn produced by special synthesis pseudo reactions from the metabolites in the network. In this second variant, the biomass reaction typically comprises only 10–20 compounds. Usually, as already mentioned above, ATP hydrolysis is integrated into (both variants of) the biomass reaction representing the ATP demand for GAM (e.g. for macromolecule polymerization). Also, the biomass reaction may not only consume but also produce metabolites, e.g. the cofactors ADP or NADP^+^ or the phosphate produced due to ATP hydrolysis for GAM. Altogether, this means that the biomass reaction can be a conglomerate of functionalities and if one wants to allow modifications of the biomass reaction one first needs to decide whether to disentangle the functionalities (like GAM) to consider them separately and whether to use the precursor/monomer or the macromolecule level.

### 2.3 Adjusting the stoichiometry of components in the biomass reaction

In the following, we assume that the measured (fixed) fluxes in Equation (5) impose an infeasible system that can be resolved by (only) adjusting the stoichiometric coefficients in the biomass reaction. The more general case that fluxes need to be adjusted in addition will be discussed afterwards.

Let C be the index set of biomass components. Each biomass component k∊C has a stoichiometric coefficient $c_{k}\neq 0$ [with unit (mmol/gDW)] in the biomass reaction. Let B be the index set of all reactions that involve any of the biomass components, but without the biomass reaction itself. The biomass reaction will be treated separately because, as an important precondition, its (growth) rate must have a fixed value μ (i.e. we assume that the growth reaction is contained in the set F of reactions with fixed rates). Modifications of the biomass components are then allowed as follows: for each biomass component k∊C, a linear equality constraint exists from Equation (1), which states that the sum of all fluxes producing and consuming this component must equal 0. With $n_{k,j}$ denoting the stoichiometric coefficient of biomass component k in reaction j, the steady-state constraints for the biomass components read:


(10) ∑j∊Bnk,jrj+ckμ=0, ∀k∊C.#

To allow the modification of coefficient $c_{k}$, a variable $\beta_{k}$ is introduced:


(11) ∑j∊Bnk,jrj+(ck+βk)μ=0⇔∑j∊Bnk,jrj+βkμ=-ckμ, ∀k∊C#

This equation also makes clear why μ must have a fixed value because otherwise the constraint would not be linear, but bilinear. To ensure that also with the modifications $\beta_{i}$, the sum of all biomass components stays constant (usually at 1 g) an additional constraint is required:


(12) ∑k∊CβkMWk=0. 

This ensures that the modifications $\beta_{k}\;$(mmol/gDW) multiplied by their molecular weights $MW_{k}$ (g/mmol) do not change the overall biomass weight. It is also reasonable to restrict $\left|\beta_{k}\right|$ to values below $\left|c_{k}\right|$ to prevent the possibility that some required biomass components are being completely replaced by others (or even produced).

We can now formulate the optimization problem for finding minimal adjustments in the biomass reaction stoichiometry to make the system feasible. Analogous to (7), when minimizing with a QP, $\beta_{k}^{2}$ can be directly used in the objective function and be multiplied with weights $z_{k}$


(13) min∑k∊Czkβk2s.t. 1–3, 5, 11,12,zk≥0

while for an LP, analogous to Equations (8) and (9), two separate variables


(14) $$\beta_{k}^{+}\geq 0,\;\beta_{k}^{-}\geq 0,\;\beta_{k}^{+}-\beta_{k}^{-}=\beta_{k}$$

are again required leading to the LP:


(15) min∑k∊Czk(βk++βk-).s.t. 1–3, 5,11,12, (14),zk≥0

Next we consider how to weight the $\beta_{k}$ in the objective function. Here, it appears reasonable to minimize relative changes, i.e. to set ${{z_{k}=1/c}_{k}}^{2}$ in the QP and $z_{k}=1/\left|c_{k}\right|\;$in the LP. This minimizes the relative molar changes but one can also consider to use the ${MW}_{i}\;$as weights to obtain a minimization of the relative changes in gram.

In some cases, adjustment of the biomass composition alone will be sufficient to resolve the infeasibility induced by inconsistent measured (fixed) fluxes in Equation (5). In general, however, it may be necessary to also adjust some of the given fluxes to obtain a feasible system. In this case, the minimizations (13)/(15) can be merged with those from (7) and (9), respectively. For example, the merged minimization for flux and biomass adjustments in the QP formulation then reads:


(16) min⁡∑i∊Fwiδi2+∑k∊Czkβk2.s.t. 1–3, 6,11,12, wi≥0,zk≥0

The weights $w_{i}$ and $z_{k}$ can be chosen as before or be adapted to reflect desired priorities in the adjustments (e.g. biomass composition before fluxes).

### 2.4 Treatment of GAM when adjusting the biomass reaction

It can be useful to treat GAM separately from the biomass composition. First of all, it should be noted that GAM does not influence the biomass weight because it corresponds to an ATP hydrolysis stoichiometry (ATP + H_2_O → ADP + P_i_ + H^+^) which is mass balanced. Having GAM integrated in the biomass reaction greatly changes the affected stoichiometric coefficients of the metabolites involved in GAM, which then mainly reflect GAM and not the mass requirements for biomass. This is particularly a problem for ATP because it is also a constituent of the RNA but in a much lower amount than in GAM. One way to deal with this situation would be to remove GAM from the biomass reaction and treat it as a separate reaction with a measured flux (the growth rate).

To simplify separate consideration of GAM without the need to modify the model, an additional slack variable $\beta_{\mathrm{GAM}}\in [-1,1]$ is introduced (which needs to be split into $\beta_{\mathrm{GAM}}=\beta_{\mathrm{GAM}}^{+}-\beta_{\mathrm{GAM}}^{-}\;$with $\beta_{\mathrm{GAM}}^{+},\beta_{\mathrm{GAM}}^{-}\in \left[0,1\right]\;$if an LP is used). With g, we denote the value of GAM (typically, the amount of ADP produced in the biomass reaction corresponds to the GAM value). For each of the five metabolites involved with GAM (ATP, ADP, H_2_O, P_i_, H^+^), the stoichiometric coefficient $c_{k}$ in the biomass [Equation (10)] is a sum of two parts, namely the amount used for GAM ($c_{k,\mathrm{GAM}}$) plus possibly (e.g. in the case of ATP) a certain amount of the metabolite consumed or produced during the actual synthesis of biomass $\left(c_{k,B}\right):\;c_{k}=c_{k,\mathrm{GAM}}+c_{k,B}.\;$The absolute value of $c_{k,\mathrm{GAM}}$ is g but its sign can be negative (ATP and H_2_O) or positive (H_2_O, P_i_, H^+^). We now allow changes in both of these coefficients but with the restriction that the change in the GAM value ($\beta_{\mathrm{GAM}}$) is identical for all five GAM metabolites. Denoting with γ the maximally allowed change of the GAM value (γ≤g), the steady-state equations of the five metabolites k involved with GAM now read:


(17) ∑j∊Bnk,jrj+(ck,B+βk)μ +(ck,GAM+γ·sign(ck,GAM)βGAM)μ=0

Here, the second term represents the adjustment of the metabolite as biomass component while the third term is the adjustment of the metabolite as GAM component. $\beta_{\mathrm{GAM}}$ can then be added to the respective objective function, possibly multiplied by some weighting factor and allows thus separate adjustment of the GAM stoichiometry.

### 2.5 Implementation in CNApy

To facilitate the application of the proposed method, it was implemented and integrated in our software CNApy (Thiele *et al.* 2022) available from github.com/cnapy-org/CNApy. The new functionality is an extension of the previously implemented function for dealing with infeasible FBA scenarios (Klamt and von Kamp 2022) and can be conveniently accessed via the GUI. In particular, the parameters for GAM adjustment can be readily set in this manner without the need for manual modification of the biomass equation.

## 3 Results

In the following, we present results when applying our new method to different metabolic models and sets of measured fluxes reported in the literature. For illustration and as a proof of principle, we first construct and solve a fictive example scenario of a biomass correction.

### 3.1 Simple example as proof of principle

We used EColiCore2 (ECC2), a core model of the metabolism of *E. coli* derived from the genome-scale model iJO1366 (Hädicke and Klamt 2017), endowed it with sum formulae for all metabolites and computed the FBA solution maximizing growth on glucose. We then changed in the biomass reaction the coefficients for two amino acids: for tyrosine, we increased the coefficient by 0.1 from 0.1379 to 0.2379 mmol/gDW. Simultaneously, we reduced the coefficient for valine by 0.1547 ensuring that the sum of all components still yields 1 g of biomass. We fixed the previously found FBA solution, which is now, with the changed stoichiometries for the two amino acids, infeasible and computed the minimal adjustments in the biomass reaction to obtain a feasible scenario. As expected, our procedure corrects the modified coefficients for the two amino acids back to the original values.

### 3.2 GAM demand of ATP in iML1515 is likely too high for anaerobic conditions

We first apply our method to data published in Monk *et al.* (2016), see Table 2. Concretely, we use the measurements of an anaerobic fermentation of *E.coli* MG1655 (mean and standard deviation of triplicates) together with the iML1515 (Monk *et al.* 2017) metabolic model. First of all, it should be noted that this dataset (amongst others) was used for the calculation of GAM/NGAM in iML1515. Looking at the elemental balance over the measured mean fluxes shows that there is carbon in excess of 5.79 mmol/(gDW h) available. Since CO_2_ production was not measured (and can thus be freely used by the model), a closed carbon balance is not to be expected but in any case enough carbon is available for all the fermentation products and the biomass. Excess carbon can also be easily compensated by many other exchange reactions for organic metabolites contained in the model that can act as additional outflows. Nonetheless, using the measured mean values for an FBA results in an infeasible system in the iML1515 model.

**Table 2.** Reported measured fluxes in *E.coli* under anaerobic conditions (Monk *et al.* 2016) and GAM values (Monk *et al.* 2017) together with the calculated adjustments of fluxes and of GAM.

	Measurement (std. dev.)/model value	A: QP flux adjustment	B: QP flux + GAM adjustment	C: QP for adjustment of fluxes including separate GAM reaction
Glucose uptake	16.69 (0.24)	16.71	16.70	16.73
Ethanol excretion	11.22 (0.6)	11.25	11.23	11.27
Acetate excretion	11.71 (1.14)	Unchanged	Unchanged	Unchanged
Lactate excretion	0 (0)	Unchanged	Unchanged	Unchanged
Formate excretion	22.17 (1.69)	22.25	22.20	22.31
Succinate excretion	1.86 (0.04)	1.862	1.861	1.863
Growth rate *μ*	0.46 (0.02)	0.261	Fixed	0.363
GAM in biomass	75.38	Fixed	29.43	Not applicable
Flux through GAM reaction^a^ (for scenario C only)	0.46 (0.02) (same as *µ*)	Not applicable	Not applicable	0.220
OV		1.998	0.846	3.373

Three different scenarios (A, B, and C) were considered for adjusting the measured rates or/and GAM values to make the system feasible. Flux rates are in [mmol/(gDW h)], the growth rate in (1/h) and GAM in (mmol/gDW). OV: objective value of the minimization.

a The GAM reaction represents an artificial reaction added in scenario C to represent the GAM demand via a mass-balanced ATP hydrolysis reaction. For further explanation, see text.

We therefore apply first our previous approach to make the system feasible [Equations (7)/(9)], without correcting the biomass reaction. Using a QP and the reciprocals of the measured standard deviations as weights, the correction procedure results in a markedly decreased growth rate, from 0.46 to 0.261/h, while the other fluxes do not change much (Table 2, scenario A). Using an LP leads to a similarly decreased growth rate of 0.260/h with the other fluxes unchanged. When only GAM adjustment (with a maximal change of 50 mmol/gDW) is allowed, which entails a fixed growth rate, again the measured fluxes hardly change but now GAM is reduced from 75.38 to 29.43 mmol/gDW (Table 2, scenario B). With an LP, GAM is reduced slightly further to 29.36 mmol/gDW with the other fluxes being unchanged.

As explained in Section 2, since GAM corresponds to a mass-balanced hydrolysis reaction that does not affect the biomass composition, it can also be taken out of the biomass reaction and be considered as an independent reaction (here 75.38 ATP + 75.38 H_2_O → 75.38 ADP + 75.38 P_i_ + 75.38 H^+^) with the same fixed rate as the measured (growth) rate of the biomass reaction. This setting, with a QP, results in a reduced growth rate together with a much greater reduction of the flux through the GAM reaction while the other fluxes are hardly affected (Table 2, scenario C). With an LP in this scenario, the rate through the GAM reaction drops further to 0.179 while all other measured rates (including the growth rate) remain unchanged. All these results show that a marked reduction in GAM alone is sufficient to make the system feasible and maintain the measured growth rate. They also highlight that changes in the substrate uptake/product excretion rates cannot contribute much to balance the system because with an LP they always remain unchanged and with a QP they undergo only minor corrections. Furthermore, allowing only corrections in the measured fluxes (scenario A), the likely overestimation of the GAM demand of ATP in the model would not have been detected.

### 3.3 Data from Boecker *et al.* (2021)

As second example, we use as a set of flux values from the data of the publication Boecker *et al.* (2021). These measurements were taken in wild-type *E.coli* under anaerobic conditions and ECC2 was again used as underlying metabolic network model. Notably, this model uses the same biomass reaction (stoichiometry) as the parent model *i*JO1366 (Hädicke and Klamt 2017). To reflect the anaerobic condition, several reactions (including the oxygen uptake) are deactivated in the network model. The measurements (see Table 3) comprise the glucose and ammonium uptake, various fermentation product excretions and the growth rate. Looking at the elemental balances of the measured rates shows that more carbon leaves the system in the form of fermentation products and biomass than enters via glucose. In total, there is a carbon shortage of 15.53 mmol/(gDW h). In contrast, nitrogen is available with an excess of 1.38 mmol/(gDW h) indicating that the percentage of nitrogen-rich compounds in the biomass in the experiment are higher than in the biomass reaction of the iJO1366 model.

**Table 3.** Measured fluxes in *E.coli* under anaerobic conditions reported in Boecker *et al.* (2021) and selected biomass stoichiometries in the modified ECC2 model together with the calculated adjustments of fluxes, GAM and biomass composition.

	Measurement (std. dev.)/model value	A: LP flux adjustment	B: LP flux + GAM adjustment	C: LP flux + GAM + biomass adjustment
Glucose uptake	13.48 (0.04)	15.63	16.25	16.24
NH_4_ uptake	6.52 (n/a)	4.18	5.14	5.39
Ethanol excretion	10.81 (0.14)	Unchanged	Unchanged	Unchanged
Acetate excretion	11.90 (0.19)	Unchanged	Unchanged	Unchanged
Lactate excretion	0.83 (0.05)	Unchanged	Unchanged	Unchanged
Formate excretion	21.88 (0.37)	Unchanged	Unchanged	Unchanged
Succinate excretion	1.77 (0.02)	Unchanged	Unchanged	Unchanged
Growth rate	0.476 (0.004)	0.386	Fixed	Fixed
GAM	31.578	Fixed	17.49	14.11
Protein	1	Fixed	Fixed	+13.45%
RNA	1	Fixed	Fixed	−15.44%
Phospholipids	1	Fixed	Fixed	−30%
OV		70.95	42.17	38.96

Three different scenarios (A, B, and C) were considered allowing different adjustments to resolve the unbalanced system. Flux rates are in [mmol/(gDW h)], the growth rate in (1/h), GAM and biomass constituents in (mmol/gDW). OV: objective value of the minimization.

For the biomass adjustment calculations, the biomass reaction of ECC2 was rearranged with the organic constituents collected into six pseudo-metabolites (protein, DNA, RNA, phospholipids, cell wall, cofactors). Each pseudo-metabolite enters with coefficient 1 into the biomass reaction and is produced by one reaction where its biomass constituents enter with their original stoichiometry (this also determines the molecular mass of the pseudo-metabolite). The reactions that produce protein, DNA, and RNA also contain the maintenance requirements as estimated in Neidhardt *et al.* (1990) which are subtracted from the total GAM of the original biomass reaction. For minimization of the modifications, an LP is used as it typically leads to a solution with less changes than a QP. The sum of relative changes is minimized, and the changes to the flux values are weighted 100 times higher than changes to the biomass reaction to force corrections in the biomass reaction with higher priority. Changes to GAM are limited to 30 mmol/gDW and changes to the stoichiometric coefficients in the biomass reaction are limited to 30%.

When only changes to the flux measurements are allowed (scenario A in Table 3) then, to make the system feasible, the glucose uptake increases while the growth rate decreases which reflects the carbon imbalance in the flux measurements. In contrast, the ammonium uptake is decreased because it is available in excess at the outset and in addition even less of it is needed due to the reduced growth rate after flux adjustment.

For the system considered in Table 3, it is not possible to make it feasible by biomass composition adjustments alone. Recall that the growth rate needs to be fixed for biomass adjustment and with the given substrate uptake/product excretion profile it is not possible to generate sufficient biomass precursors together with the ATP demanded by GAM. In fact, when excluding the ammonium and growth rate measurements, ECC2 achieves a growth rate of at most 0.11/h. When changes to flux rates and GAM are allowed, a reduction of GAM together with increased substrate uptake is needed to make the scenario feasible (scenario B in Table 3). In fact, the glucose uptake increases even further than with flux changes alone because now the growth rate cannot be lowered any more. When all biomass adjustments together with flux changes are enabled (scenario C) then the situation is similar as before, but somewhat less substrate is needed while GAM is decreased further. In addition 30% of the phospholipids together with 15.44% of the RNA are replaced by 13.45% more protein. This has the effect that in the new biomass, the ratio of N to C is increased so that the ammonium uptake flux needs to be decreased less than in the situation without biomass composition adjustment. Altogether, biomass composition adjustment somewhat reduces the amount of flux changes necessary to balance the system.

### 3.4 Application guideline

This section offers a guideline (workflow) how to use the different adjustment procedures in practice [it extends the guideline given in section 3.5 in Klamt and von Kamp (2022)]. We are given a metabolic model and some measured (or known) fluxes. We assume that the model without adding the measured fluxes as constraints is feasible, i.e. an FBA with any objective function finds a feasible solution, while it becomes infeasible when adding these constraints.

#### 3.4.1 Step 0: preparation and preliminary analysis

Run a flux variability analysis (FVA) of the model (without fixing the measured rates) and check whether the measured rates are within the calculated ranges. If not, adjust reaction bounds (e.g. increase the substrate uptake limit) so that the FVA ranges include your measurements. If the model including the fixed measured rates is now feasible you can stop here. Next, check whether the model contains any fixed demands. Typically, nongrowth-associated maintenance (NGAM) will be present in the form of an ATP hydrolysis reaction with a lower bound greater zero. Consider if the fixed demands are appropriate for your situation and if necessary relax or remove them. Also, a fixed demand could be removed and then treated as a measurement which would make it adjustable. As an example, the lower bound of the NGAM reaction could be set to zero and the original lower bound be used as measurement value to make NGAM adjustable.

In models with correct chemical formulas for all metabolites involved in the given fluxes and biomass reaction: check the elemental balances to get an overview over gross imbalances. This reveals whether the substrate contains sufficient elemental matter for all measured products.

In the following, three steps for the application of the adjustment procedure are described. We recommend to execute all steps to get a comprehensive overview over the possible reason(s) for the infeasibility and to identify the most plausible solution.

#### 3.4.2 Step 1: make system feasible using flux corrections only

Since we assumed that the network (without measured fluxes) has a feasible solution using corrections of measured fluxes (alone or in conjunction with biomass adjustments) will always give a feasible solution. Decide for a weighting scheme [see section 3.5 in Klamt and von Kamp (2022) for some general rules]. Compare the LP and QP solutions. The LP solution is not necessarily unique, while the QP solution will distribute the (weighted) corrections as evenly as possible. If the QP solution adjusts some fluxes much more than others this is a good indication where the main problem lies, otherwise the LP solution is preferable.

Also check whether the corrected fluxes lie on the reaction bounds. In those cases, it may be useful to relax these bounds beforehand to enable a wider range of adjustments.

#### 3.4.3 Step 2: make system feasible using biomass reaction adjustments only

Biomass adjustments alone may not always give a feasible solution, for instance, if the elemental imbalances are too large.

(a) Use GAM adjustment alone, if applicable to the biomass reaction of your model. For this, it may be useful to increase the maximal change in GAM ATP amount to the actual GAM value of the model. If this makes the system feasible, this indicates that the model GAM may be too high for your situation.
(b) Use adjustment of biomass components alone, here it may be useful to increase the amount of relative coefficient change.
(c) Use GAM and biomass component adjustments together to see if this is sufficient to make your system feasible.

#### 3.4.4 Step 3: enable flux corrections and biomass adjustments

Allow simultaneous adjustments of fluxes and biomass reaction and compare with the result of step 1 to see to what degree biomass reaction adjustments reduce the amount of flux corrections necessary to make the system feasible. If there is, e.g. insufficient substrate uptake then biomass composition adjustments may have little impact on making the system feasible.

When using multiple adjustment options consider how they will be weighed against each other. The cost of GAM adjustment can be changed directly with a weighting factor. The cost of flux corrections are multiplied with the reciprocal value of a default scale factor so that by lowering this factor, the cost of flux corrections is increased.

The results of steps 1–3 should be compared and the most plausible corrections chosen as final result.

## 4 Discussion

Starting from a previously presented framework for finding minimal adjustments of measured fluxes to make an infeasible FBA scenario feasible (Klamt and von Kamp 2022), we further developed this framework to also allow adjustments in the biomass composition. Such an extension appears reasonable and useful because the stoichiometry of the biomass synthesis reaction in FBA models is often a rough estimation and a source of high uncertainty as it varies under different conditions. We have implemented the new methods in our software tool CNApy allowing its application in a user-friendly environment.

It should be noted that our method differs from a related tool, BOFdat (Lachance *et al.* 2019), which has been specifically developed to for the initial derivation of the stoichiometry of the biomass reaction for a given model/species, requiring various measurements and datasets (including macromolecular weight fractions, sequence data, proteomic data, etc.). In contrast, our method seeks to derive (smallest) modifications of a given biomass reaction to reach consistency in a specific scenario with available flux measurements.

Applying this framework to datasets with measured fluxes of *E.coli* grown under anaerobic conditions, we could draw important conclusions. The example with measurements of Monk *et al.* (2016) analyzed with the iML1515 model showed that the measurements under anaerobic conditions can be made compatible with the model by drastically reducing GAM alone whereas modifications of the substrate uptake/fermentation product excretion rates can hardly contribute to equilibrating the system. In addition, trying to limit the reduction of GAM can only be compensated by a reduced growth rate which effectively also reduces the amount of ATP used for growth. Hence, this example highlights that a change of the GAM value implicitly contained in the biomass reaction may be more appropriate than adjusting fluxes to resolve infeasibility.

The results of this example confirm the findings of Dinh *et al.* (2022) showing that the values for GAM used in the biomass reaction may have profound effects on computed metabolic fluxes in FBA studies. Moreover, it also motivates a reconsideration of the way in which GAM is derived from measurements when configuring a metabolic model. Recall that the model without maintenance ATP (i.e. GAM and NGAM set to zero) is used to calculate the maximum amount of ATP that can be produced under a set of measurements. Naturally, only such measurements are used where the substrate uptake and all relevant fermentation product excretion rates together with the growth rate have been accurately determined. However, a first problem arises because for aerobic conditions the calculated maximum surplus ATP production rate depends on the P/O ratio that is inherent in the way the respiratory chain has been modeled. In reality, the detailed mechanism of the respiratory chain with its many components has been notoriously difficult to ascertain and the effective P/O ratio is known to be variable (Ferguson 2010, Wikström and Springett 2020, Dunn 2023). Therefore, a fixed (optimistic) P/O ratio may very well be an overestimation of the actual P/O in certain situations. To make matters worse, alternative oxidases, which reduce the P/O ratio of respiration to allegedly reduce oxidative stress, are present in many eukaryotes and have also been identified in some bacteria (Dunn 2023).

In a similar vein, it is hardly possible to verify that the maximum surplus ATP rate can be realized because it may depend on certain other flux rates that are above the capacity of what can be realized in the organism. This is a general problem of FBA as it tends to overestimate the objective flux (e.g. growth or net ATP production rate) because regulatory, thermodynamic, and other constraints are not being taken into account, although recent developments seek to integrate further constraints, such as enzyme capacity constraints (Sánchez *et al.* 2017, Bekiaris and Klamt 2020), to better reflect such natural limitations.

Apart from this, it is also not clear that all surplus ATP, independent of whether it comes from respiration or other processes, is being used for GAM or NGAM (Farmer and Jones 1976). Potential surplus ATP could also be hydrolyzed by futile cycles which are, as far as the network structure is concerned, present in most metabolic networks. Evidence that one such futile cycle can actually operate in *E.coli* has been reported in Yang *et al.* (2003), with the amount of ATP being wasted higher at low growth rate. Such a type of process is then an ATP demand that fits neither the NGAM (fixed demand) nor the GAM (demand increases with growth rate) logic.

Another point is that the growth condition under which measurements have been made may have an impact on the amount of surplus ATP as calculated by the model. An indication that this could be the case can be seen in Supplementary Figure 7 of Monk *et al.* (2017), because there the ATP production rates for different growth conditions tend to cluster together.

All these reasons and the varying values in Table 1 underline that the GAM (and NGAM) values are affected by a high degree of uncertainty. In particular, GAM determined from an aerobic experiment may very well be too high for a comparable anaerobic situation. The way (N)GAM has been determined for iML1515 [Supplementary Figure 7 of Monk *et al.* (2017)] entails that, when using the measured mean values from the experiments below the GAM line in this figure, the FBA will become infeasible. Therefore, it is useful to have a method at hand with which one can quickly check if such an infeasibility could be resolved by reducing the model GAM.

The second realistic scenario with the ECC2 example again shows that the GAM value, which was directly taken from its parent model iJO1366, is probably too high for an anaerobic situation. Here we also allowed biomass composition adjustment to illustrate how this can be used to derive suggestions about biomass changes that can reduce the amount of flux changes necessary to make the system feasible. It should be stressed that the changes calculated here are only a rough suggestion because, without additional constraints (e.g. measurements of uptake of phosphate or sulfur or release of CO_2_), there remains a relatively large degree of freedom to make adjustments in the biomass composition (note that as many slack variables are introduced as there are components in the biomass reaction). Nonetheless, it is possible to get an idea to which degree biomass adjustments can reduce necessary changes to the fluxes to balance an infeasible system. In this example, the effect is rather small which can be expected because the original flux measurements already show a significant elemental imbalance. To get better and more constrained results, it would be useful to include additional information about the real biomass composition as, for instance, its elemental composition. If the elemental composition was available, it could be integrated via additional constraints into the LP/QP. Flux estimates obtained from isotopic tracer experiments could also further decrease the degrees of freedom.

Here, we have concentrated on the case that only one biomass reaction is being used in the FBA which currently is the typical case for constraint-based models. When multiple biomass compositions (derived from different environmental conditions) become available it becomes possible to use these to predict the biomass composition under a new condition. First methods in this direction have been proposed in Schulz *et al.* (2021) (using artificially created multiple biomass reactions) and may in future complement our methods for dealing with FBA infeasibilities.

## Data Availability

The software CNApy is available from https://github.com/cnapy-org/CNApy. Models and CNApy scenario files that contain the experimental measurements used herein can be downloaded from https://github.com/cnapy-org/CNApy-projects/releases (iML1515_biomass_adjustment.zip for the iML1515 example andECC2_biomass_adjustment.zip for the two ECC2 examples, respectively).
